# The Dynamic DNA Demethylation during Postnatal Neuronal Development and Neural Stem Cell Differentiation

**DOI:** 10.1155/2018/2186301

**Published:** 2018-03-11

**Authors:** Huikang Tao, Pei Xie, Yuhang Cao, Liqi Shu, Liping Li, Junchen Chen, Guangfeng Tian, Yingliang Zhuang, Qiang Shu, Xuekun Li

**Affiliations:** ^1^The Children's Hospital, School of Medicine, Zhejiang University, Hangzhou 310058, China; ^2^Institute of Translational Medicine, School of Medicine, Zhejiang University, Hangzhou 310029, China; ^3^School of Medicine and Health Sciences, George Washington University, Washington, DC 20037, USA

## Abstract

**Background:**

DNA demethylation, the conversion of 5-methylcytosine (5mC) to 5-hydroxymethylcytosine (5hmC), 5-formylcytosine (5fC), and 5-carboxylcytosine (5caC), plays important roles in diverse biological processes and multiple diseases by regulating gene expression.

**Methods:**

In this study, utilizing DNA dot blot, immunofluorescence staining, and qRT-PCR, we studied the expression pattern of Tets, the enzymes governing DNA demethylation, and the levels of 5hmC, 5fC, and 5caC during the postnatal neuronal development of mice.

**Results:**

It was found that 5hmC, 5fC, and 5caC were highly enriched in multiple brain regions and aNSCs and displayed temporal and spatial patterns during postnatal neuronal development and the differentiation of aNSCs. Consistently, the expression of *Tets* also exhibited temporal and spatial patterns.

**Conclusion:**

DNA demethylation displayed dynamic features during postnatal neuronal development and the differentiation of aNSCs of mice, which could contribute to appropriate gene expression.

## 1. Introduction

In mammalians, DNA methylation at the fifth carbon of cytosine (5-methylcytosine, 5mC), the most intensively studied DNA modification, plays pivotal roles in multiple biological processes including chromatin structure, gene imprinting, X chromosome inactivation, and genomic stability; embryonic and postnatal development; ageing; and diseases by regulating gene expression [1–4]. It has been uncovered that 5mC can be oxidized to 5-hydroxymethylcytosine (5hmC), an active DNA demethylation process in mammalians, by ten-eleven translocation (Tet) dioxygenases (Tet1, 2, and 3) [5, 6]. Besides, 5hmC can be further oxidized to 5-formylcytosine (5fC) and 5-carboxylcytosine (5caC) by *Tets* [5, 7]. Both 5fC and 5caC can be targeted by thymine DNA glycosylase (TDG) and subsequently processed through the base excision repair (BER) DNA repair pathway, and they generated an unmodified cytosine [8, 9]. Mounting evidences indicate that 5hmC-mediated epigenetic modification plays critical roles in neuronal development and function, and the aberrant DNA demethylation is associated with neurological disorders including Rett syndrome, autism, Alzheimer's disease, Huntington's disease, and FXTAS [7, 10–14].

Previous studies have found that 5hmC widely exists in mammalian tissues, displays a cell-/tissue-specific distribution pattern, and is abundant in neural tissues [7, 14–18]. Further, 5hmC also exhibits dynamic features during embryonic and postnatal neuronal development [7, 14, 18, 19]. However, the distribution pattern of 5fC and 5caC during postnatal neuronal development and adult neural stem cell (aNSC) differentiation remains largely unknown. Meanwhile, the expression of *Tets* during postnatal neuronal development is also unclear. In the present study, we studied the distribution patterns of 5hmC, 5fC, and 5caC in the postnatal mouse brain, cultured adult neural stem cells (aNSCs) with immunostaining and dot blot, and compared their global levels. We found that 5hmC, 5fC, and 5caC were all detectable in neuronal cells in multiple brain regions. During the postnatal neuronal development, the global levels of 5hmC increased in all these three brain regions, whereas 5fC did not show a significant change. 5caC significantly decreased in the cortex while the alteration is slight in the hippocampus and cerebellum. QRT-PCR results showed that the mRNA levels of *Tets* were decreased in the cortex during postnatal neuronal development, and *Tet2* had the highest expression level in the cortex and hippocampus. Further, 5hmC, 5fC, and 5caC were all detected in aNSCs and the global levels also changed during aNSC differentiation. Our results indicated the existence of 5hmC, 5fC, and 5caC in the brain and aNSCs, and *Tets* exhibited dynamic expression patterns during the postnatal neuronal development.

## 2. Materials and Methods

### 2.1. Animals and Tissue Preparation

C57BL/6 male mice and pregnant mice were purchased from the Shanghai Experimental Animal Center (Shanghai, China). The generation of *Tet1* and *Tet2* mice was described as previously [20, 21]. The animals were housed in the animal center of Zhejiang University on a 12 : 12 light/dark cycle with free access to food and water. All experimental procedures were performed according to protocols approved by the Animal Care and Use Committee of Zhejiang University. In this study, the day of birth was considered as postnatal day 1 (P1), 14 days after the birth as P14, and 8 weeks as adult.

Mice were deeply anesthetized with chloral hydrate (50 mg/kg, i.p.) and transcardially perfused with cold phosphate-buffered saline (PBS) followed by perfusion of 4% paraformaldehyde (PFA). The brains were gently removed and postfixed in 4% PFA overnight at 4°C. On the second day, the brain samples were transferred into 30% sucrose solution for dehydration at 4°C until they settled down in the solution. The brain samples were embedded in OCT (Thermo Fisher Scientific) and sectioned in the coronal plane (20 *μ*m) with a cryostat (Leka). Sections were collected into the cryoprotectant solution and stored at −20°C until further processing.

### 2.2. The Isolation, Proliferation, and Differentiation Assays of NSCs

After being deeply anesthetized with chloral hydrate (50 mg/kg, i.p.), adult male mice were sacrificed and brains were removed and put into cold PBS. ANSCs were isolated from the forebrain as described previously [22] and cultured with DMEM/F-12 medium (DM-25, Omega Scientific) containing 20 ng/ml basic fibroblast growth factor (FGF-2, PeproTech), 20 ng/ml epidermal growth factor (EGF, PeproTech), 2% B27 supplement (Gibco), 1% penicillin-streptomycin (Gibco), and 2 mM L-glutamine (Gibco) in a 5% CO_2_ incubator at 37°C.

For the proliferation assay of aNSCs, aNSCs were plated onto coverslips (BD Biosciences) with fresh medium. 5-Bromo-2′-deoxyuridine (BrdU, Sigma-Aldrich) was added into the culture medium at the final concentration of 5 *μ*M for 8 h. ANSCs were then washed with PBS and fixed with 4% PFA for 30 min at room temperature. For the differentiation assay, aNSCs were treated with 5 *μ*M forskolin (Sigma-Aldrich) and 1 *μ*M retinoic acid (Sigma-Aldrich) for 2 days and then fixed with 4% PFA for 30 min at room temperature.

### 2.3. 5hmC, 5fC, and 5caC Immunofluorescence Staining and Imaging

Brain sections or cell samples were washed with PBS for 30 min and treated with 1 M HCl for 30 min at 37°C followed by RNase A treatment. After being washed with PBS for 15 min, samples were blocked with PBS containing 3% normal goat serum (Vector Laboratories) and 0.1% Triton X-100 (Sigma-Aldrich) for 1 h at room temperature and then incubated with primary antibodies. The primary antibodies used for immunofluorescence were as follows: the polyclonal rabbit antibodies anti-5-hydroxymethylcytosine (Active Motif), anti-5-formylcytosine (Active Motif), and anti-5-carboxylcytosine (Active Motif); the mouse monoclonal antibody anti-NeuN (Millipore); the rabbit polyclonal antibody anti-Sox2 (Millipore); the mouse monoclonal antibody anti-Nestin (BD Biosciences); the mouse monoclonal antibody anti-*β* III-tubulin (Promega); the rat monoclonal antibody anti-BrdU (Abcam); and the rabbit polyclonal antibody anti-glial fibrillary acidic protein (Dako). On the second day, samples were washed with PBS for 30 min and then incubated with secondary antibodies Alexa Fluor 488 goat anti-rabbit IgG, Alexa Fluor 568 goat anti-mouse IgG, and Alexa Fluor 568 goat anti-rat IgG (Invitrogen). DNA was counterstained with 4′-6-diamidino-2-phenylindole (DAPI, Sigma-Aldrich). After final washes, sections were mounted onto glass slides and coverslipped with mounting medium (Vector Laboratories). All immunostaining experiments were repeated with the sections from at least three animals of each age. Fluorescence images were viewed and captured with a Zeiss confocal microscope.

### 2.4. Genomic DNA Isolation and Dot Blot

Genomic DNA was extracted as described previously [7]. Briefly, the dissected brain samples or cells were homogenized in lysis buffer (5 mM EDTA, 0.2% SDS, 200 mM NaCl in 100 mM Tris-HCl, pH 8.5) supplemented with proteinase K (Sigma-Aldrich) and were digested at 56°C overnight. On the second day, the samples were treated with RNase A for at least 12 h at 37°C. An equal volume of phenol : chloroform : isoamyl alcohol (25 : 24 : 1, Sigma-Aldrich) was added, mixed completely, and centrifuged at 14,000 rpm for 10 min. An equal volume of isopropanol was added to the supernatant to precipitate DNA, which was dissolved with 10 mM Tris-HCl (pH 8.0). The concentration of DNA was quantified with NanoDrop (Thermo Fisher Scientific).

Dot blot was performed as described previously [7, 23]. Briefly, DNA samples were denatured and spotted onto the membrane (Amersham Biosciences) on a Bio-Dot apparatus (Bio-Rad). After being heated in a hybridization oven for 30 min at 80°C, the sample membrane was blocked with 5% fat-free milk in Tris-buffered saline (TBS) for 1 h before incubation with the primary antibody at 4°C overnight. The following primary antibodies were used for dot blot: the polyclonal rabbit antibodies anti-5-hydroxymethylcytosine (Active Motif), anti-5-formylcytosine (Active Motif), and anti-5-carboxylcytosine (Active Motif). On the second day, the sample membranes were washed with TBS for 30 min and incubated with anti-rabbit horseradish-peroxidase-conjugated secondary antibody for 30 min at room temperature. After washing with TBS for 30 min, the chemiluminescence signals were visualized with the Tanon detection system (Tanon). The signal intensities of 5hmC, 5fC, and 5caC were quantified with the Adobe Photoshop software.

### 2.5. RNA Isolation and Real-Time PCR

Total RNA was extracted by the TRIzol reagent (Invitrogen), and 1 *μ*g total RNA was used for reverse transcription according to the manufacturer's protocol (Life Technologies). The relative gene expression levels were measured by SYBR qPCR master mix (Life Technologies) with Applied Biosystems Viia 7. The following PCR primers were used: *Tet1*-F 5′-AGGGCCAAAATGAAGCAGAA-3′ and *Tet1*-R 5′-GAGGCTGATGAAAAGCTCTTAGTGT-3′, *Tet2*-F 5′-GGCAAATGTGAAGGATGCAA-3′ and *Tet2*-R 5′-CCAGCTCCTAGATGGGTATAATAAGG-3′, and *Tet3*-F 5′-CGCCTCACGGGAGACAAT-3′ and *Tet3*-R 5′-AGTGGCCAGATCCTGAAAGCT-3′. *18S* was used for internal control: forward 5′-CGGCTACCACATCCAAGGAA-3′ and reverse 5′-CCTGTATTGTTATTTTTCGTCACTACCT-3′. The PCR program starts with the denaturation step of 10 min at 95°C, followed by 40 cycles of 15 sec at 95°C and 1 min at 60°C. The relative expression levels were then calculated using the 2^−∆∆CT^ method.

### 2.6. Statistical Analysis

All statistical analysis was performed with the GraphPad Prism software. Data were presented as mean ± SEM. For comparisons between two groups, a two-tailed unpaired Student *t*-test was used. For multiple group comparisons, a one-way ANOVA followed by Tukey post hoc test was used. *p* < 0.05 was considered statistically significant.

## 3. Results

### 3.1. The Immunofluorescence Staining of 5hmC, 5fC, and 5caC in Brain Sections

**Figure 1:**
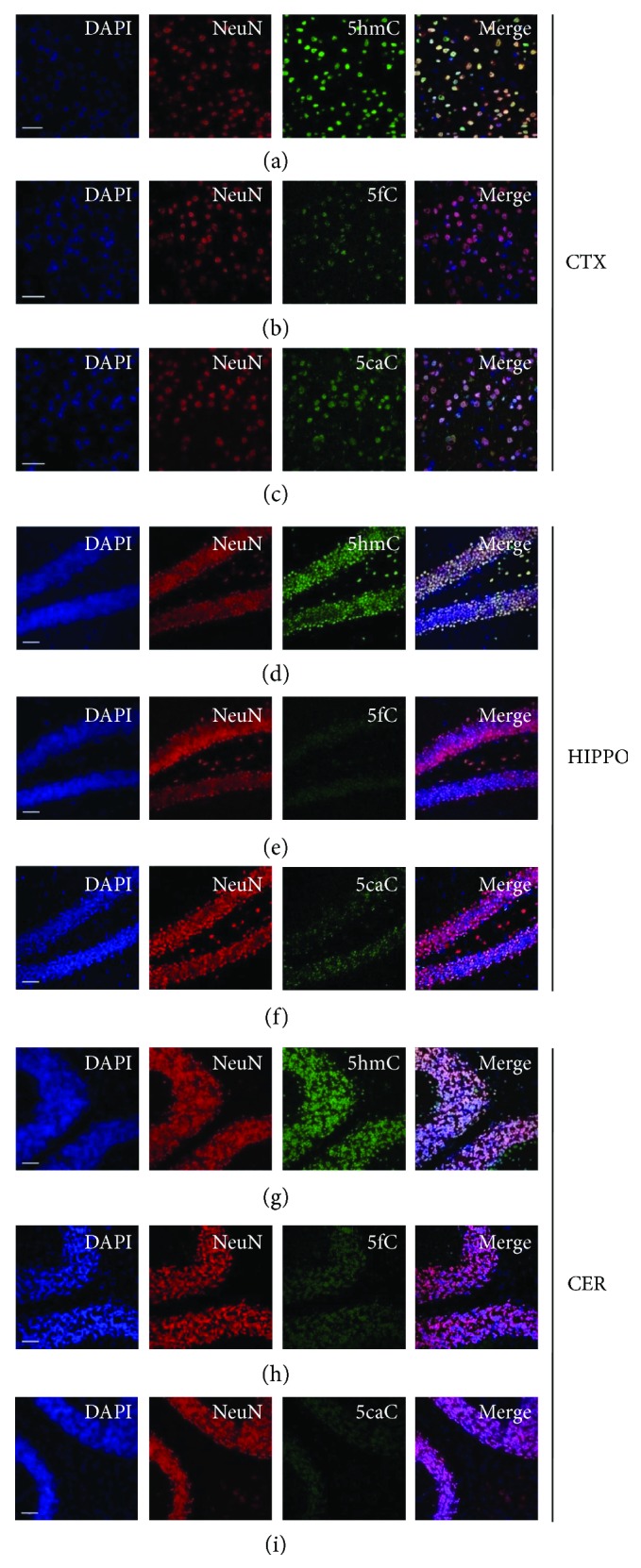
The representative immunofluorescence images of 5hmC, 5fC, and 5caC in the cortex, hippocampus, and cerebellum. Immunofluorescence of 5hmC (a), 5fC (b), and 5caC (c) in the 8 w cortex. Immunofluorescence of 5hmC (d), 5fC (e), and 5caC (f) in the 8 w hippocampus. Immunofluorescence of 5hmC (g), 5fC (h), and 5caC (i) in the 8 w cerebellum. Scale bar, 200 *μ*m.

To detect 5hmC, 5fC, and 5caC in the brain, we first performed immunofluorescence using 5hmC-, 5fC-, or 5caC-specific antibodies together with a neuronal cell marker NeuN antibody. It was found that nearly all neuronal cells (NeuN^+^) were also positive for 5hmC labeling in the cortex (Figure 1(a)), hippocampus (Figure 1(d)), and cerebellum (Figure 1(g)). Moreover, 5fC and 5caC were also detected in neuronal cells in those brain regions (Figures 1(b), 1(c), 1(e), 1(f), 1(h), and 1(i)). Taken together, these results indicated that active DNA demethylation (5hmC, 5fC, and 5caC) occurred in the genome of neuronal cells in different brain regions.

### 3.2. The Quantification of the Global Levels of 5hmC, 5fC, and 5caC in Different Brain Regions

**Figure 2:**
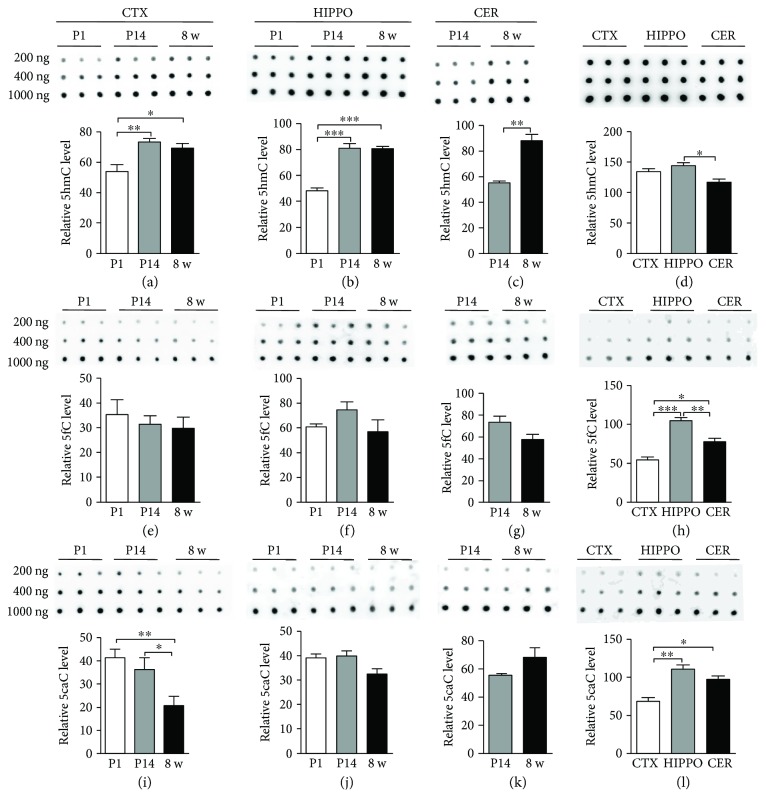
5hmC, 5fC, and 5caC in different brain regions using DNA dot blot. The relative levels of 5hmC, 5fC, and 5caC in the P1, P14, and 8 w cortex ((a), (e), (i)), hippocampus ((b), (f), (j)), and cerebellum ((c), (g), (k)). Comparison of 5hmC (d), 5fC (h), and 5caC (l) levels between different brain regions at 8 w. Data are represented as mean ± SEM (*n* = 3). Statistically significant differences were indicated: ^∗^ *p* < 0.05, ^∗∗^ *p* < 0.01, and ^∗∗∗^ *p* < 0.001.

Considering that 5hmC, 5fC, and 5caC were detectable in multiple brain regions, we next performed DNA dot blot to quantify the global levels of 5hmC, 5fC, and 5caC in those brain regions during the postnatal development (postnatal day 1 (P1), day 14 (P14), and adult (8 w)). Dot blot results showed that the 5hmC level significantly increased from P1 to P14 in the cortex and hippocampus (Figures 2(a) and 2(b)). Of note, from P14 to adult, the 5hmC level maintained stably in the cortex and hippocampus, but significantly increased in the cerebellum (Figures 2(a)–2(c)). The 5fC level did not display a significant change in those three brain regions during the postnatal neuronal development (Figures 2(e)–2(g)). Further, the 5caC level in the cortex significantly decreased in adults compared to P1 and P14 (Figure 2(i)), but it did not alter significantly in the hippocampus and cerebellum (Figures 2(j) and 2(k)).

We further compared the 5hmC, 5fC, and 5caC levels in different brain regions at adult age. The 5hmC level was similar between the cortex and hippocampus, but higher than that of the cerebellum (Figure 2(d)). 5fC and 5caC were significantly enriched in the hippocampus compared to the cortex and cerebellum (Figures 2(h) and 2(l)). Taken together, these results suggest that DNA demethylation exhibited a temporal and spatial feature during the postnatal neuronal development.

### 3.3. The Expression of Tets in Different Brain Regions by qRT-PCR

**Figure 3:**
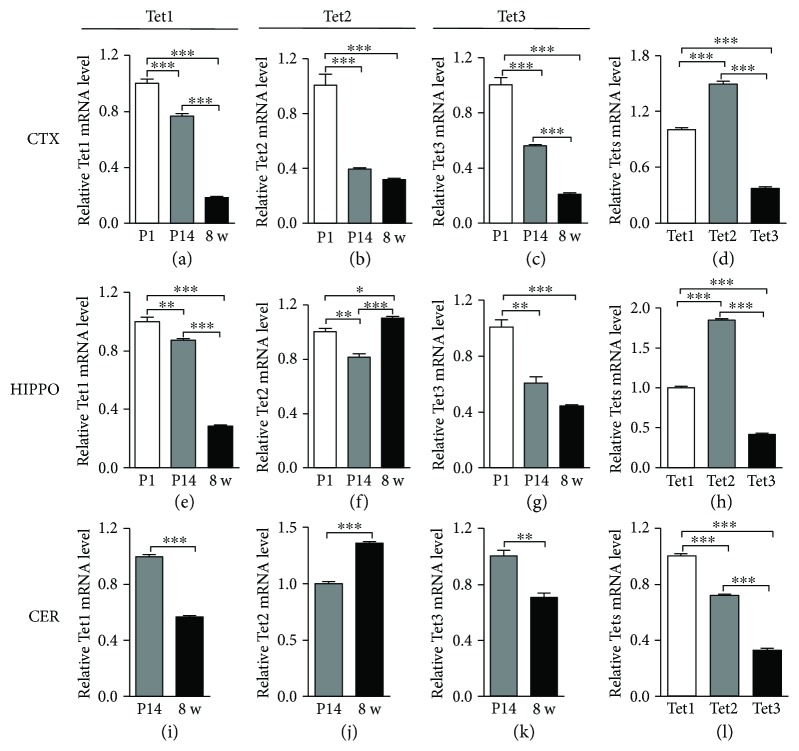
The mRNA levels of Tets in different brain regions. The relative mRNA levels of *Tet1*, *Tet2*, and *Tet3* in the cortex from P1 to 8 w ((a)–(c)). The relative mRNA levels of *Tet1*, *Tet2*, and *Tet3* in the hippocampus from P1 to 8 w ((e)–(g)). The relative mRNA levels of *Tet1*, *Tet2*, and *Tet3* in the cerebellum from P1 to 8 w ((i)–(k)). Comparison of *Tet1*, *Tet2*, and *Tet3* mRNA levels in different brain regions at 8 w ((d), (h), (l)). Data are represented as mean ± SEM (*n* = 3). Statistically significant differences are indicated: ^∗^ *p* < 0.05, ^∗∗^ *p* < 0.01, and ^∗∗∗^ *p* < 0.001.

In mammalian brain, DNA demethylation was catalyzed by Tet family proteins. We next studied the expression of *Tets* by qRT-PCR in those three brain regions from P1 to adult. It was found that the mRNA levels of *Tet1*, *Tet2*, and *Tet3* decreased from P1 to adult in the cortex (Figures 3(a)–3(c)). In the hippocampus and cerebellum, the mRNA levels of *Tet1* and *Tet3* decreased during postnatal neuronal development while *Tet2* increased (Figures 3(e)–3(g) and 3(i)–3(k)). We then compared the expressions of *Tet1*, *Tet2*, and *Tet3* in different brain regions at adult age. It was found that *Tet2* had the highest expression level in the cortex and hippocampus compared to *Tet1* and *Tet3* (Figures 3(d) and 3(h)); however, the expression of *Tet1* was the highest in the cerebellum (Figure 3(l)). These results suggested that the expression of *Tets* also displays dynamic features during the postnatal neuronal development.

**Figure 4:**
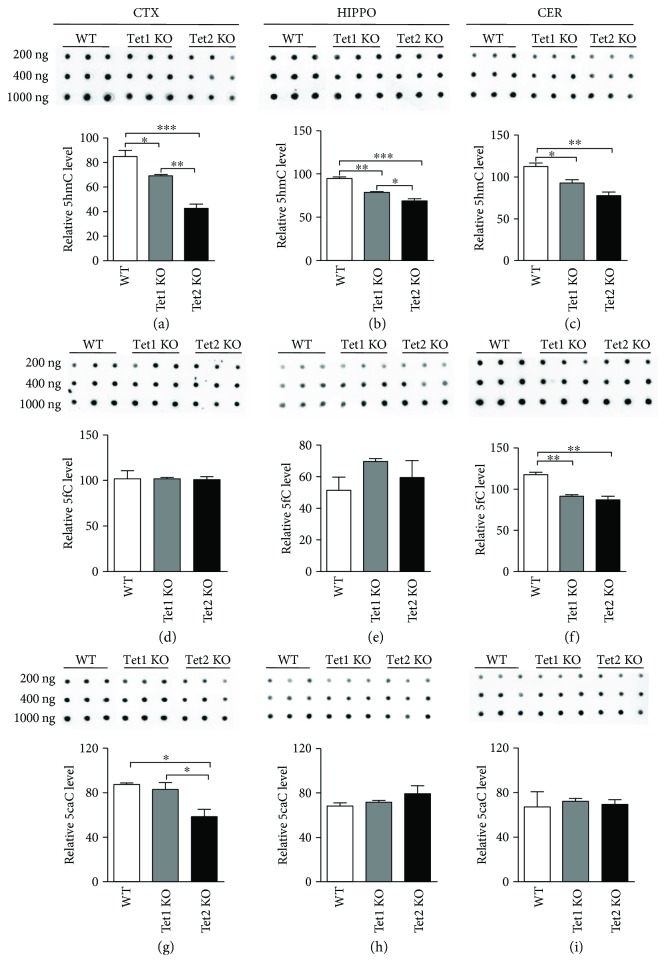
5hmC, 5fC, and 5caC in *Tet1* and *Tet2* knockout brain using DNA dot blot. The levels of 5hmC, 5fC, and 5caC in the cortex ((a), (d), (g)), hippocampus ((b), (e), (h)), and cerebellum ((c), (f), (i)) of adult *Tet1* and *Tet2* KO mice. Data are represented as mean ± SEM (*n* = 3). Statistically significant differences are indicated: ^∗^ *p* < 0.05, ^∗∗^ *p* < 0.01, and ^∗∗∗^ *p* < 0.001.

To reveal which Tet plays dominant roles in DNA demethylation during the postnatal neuronal development, *Tet1* and *Tet2* knockout (KO) mice were adopted and genomic DNA was extracted from the cortex, hippocampus, and cerebellum regions, respectively. Dot blot results showed that *Tet1* and *Tet2* KO could significantly decrease 5hmC levels in those three brain regions of mice. *Tet2* depletion displayed more dramatic effects on 5hmC levels compared to *Tet1* depletion (Figures 4(a)–4(c)). For 5fC and 5caC, *Tet1* and *Tet2* depletion showed a distinct effect in different regions. The 5fC level was decreased in the cerebellum induced by the depletion of *Tet1* and *Tet2* while no significant change was observed in the cortex and hippocampus (Figures 4(d)–4(f)). The 5caC level decreased in the cortex while there was no observable change in the hippocampus and cerebellum (Figures 4(g)–4(i)). These results suggested that Tet1 and *Tet2* both catalyzed DNA demethylation, and *Tet2* showed a more dominant effect.

### 3.4. DNA Demethylation in Cultured Adult Neural Stem Cells

To further characterize DNA demethylation in neuronal development, we isolated neural stem cells (aNSCs) from the brain of adult mice. More than 97% of the cultured aNSCs were positive for NSC markers Nestin and Sox2 (Supplemental Figures 1(a)–(d)), suggesting the homogeneity of aNSCs. The cultured aNSCs could incorporate BrdU during proliferation (Supplemental Figures 1(e)–(g)) and generate neurons and astrocytes upon differentiation (Supplemental Figures 1(h)–(k)), suggesting their capabilities of self-renewal and multipotency.

**Figure 5:**
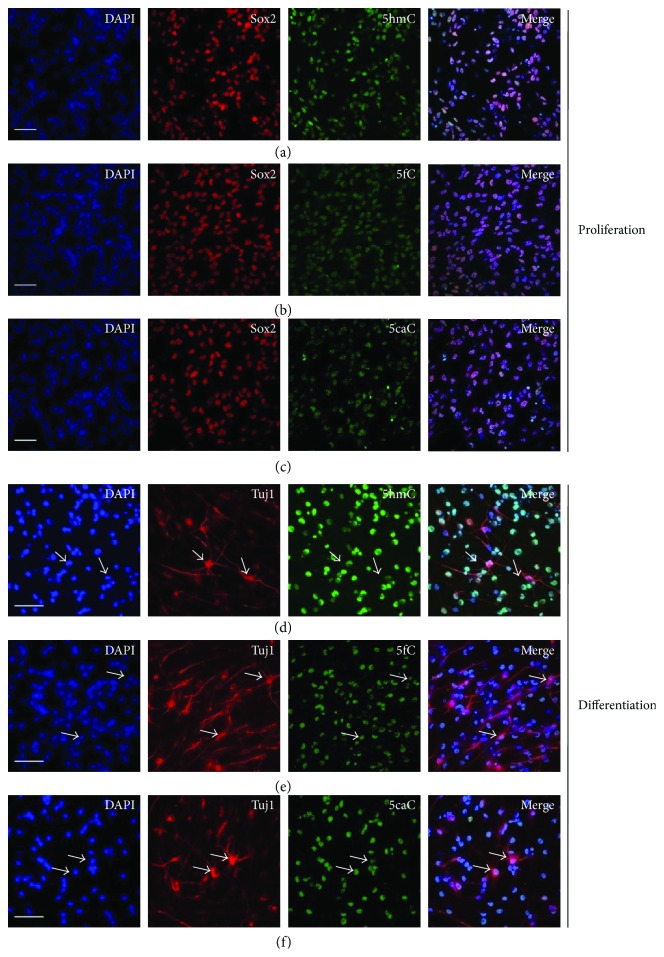
The representative immunofluorescence images of 5hmC, 5fC, and 5caC in cultured aNSCs. Immunofluorescence of 5hmC (a), 5fC (b), and 5caC (c) in cultured aNSCs. Neuronal cells generated from aNSCs were positive for 5hmC (d), 5fC (e), and 5caC (f). Scale bar, 200 *μ*m.

**Figure 6:**
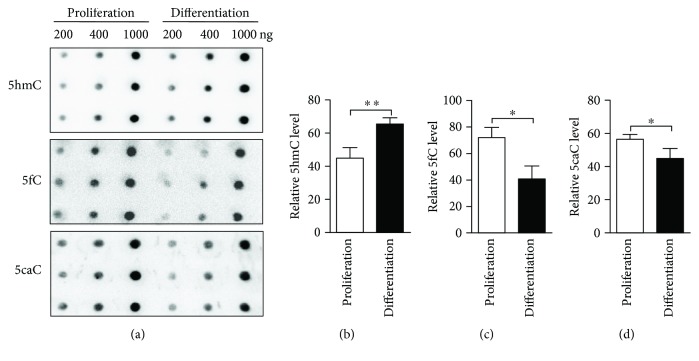
DNA demethylation in cultured aNSCs. DNA dot blots of 5hmC, 5fC, and 5caC in proliferating and differentiated aNSCs (a). The quantification of dot blots of 5hmC, 5fC, and 5caC, respectively ((c)–(d)). Statistically significant differences are indicated: ^∗^ *p* < 0.05 and ^∗∗^ *p* < 0.01.

Next, we performed immunofluorescence staining and found that 5hmC, 5fC, and 5caC were well colocalized with aNSC marker Sox2 at the proliferating condition (Figures 5(a)–5(c)). 5hmC, 5fC, and 5caC could be detected in the nuclei of neuronal cells (Tuj1^+^) generated during aNSC differentiation (Figures 5(d)–5(f)). Dot blot results showed that 5hmC, 5fC, and 5caC were all detectable in the genomic DNA of aNSCs (Figure 6(a)). 5hmC significantly increased while 5fC and 5caC decreased from proliferation to differentiation of aNSCs (Figures 6(b)–6(d)). Together, these results indicated that DNA demethylation exhibited dynamic features during the differentiation of aNSCs.

## 4. Discussion

In the present study, we detected and compared DNA demethylation in multiple brain regions and aNSCs. Our results indicated that 5hmC-, 5fC-, and 5caC-mediated DNA demethylation actively occurred not only in the brain during postnatal development but also in aNSCs. 5hmC was acquired in all three brain regions during neuronal maturation, while 5fC and 5caC displayed slight changes in the hippocampus and cerebellum but a significant decrease in the cortex. The mRNA levels of *Tet1*, *Tet2*, and *Tet3* were decreased overall in all three brain regions during postnatal neuronal development, and *Tet2* had the highest expression level in the cortex and hippocampus. We also found that 5hmC, 5fC, and 5caC existed in the genome of aNSCs, and the global level of 5hmC was increased while 5fC and 5caC were successively decreased in aNSCs. Our study showed the features of the temporospatial-specific alteration of 5hmC, 5fC, and 5caC in mammalian brain and aNSCs.

Previous studies had indicated that DNA demethylation played essential roles in neuronal development, learning and cognitive ability, and neurological disorders [7, 11, 19, 24–27]. 5hmC was acquired during embryonic and postnatal brain development and highly enriched in neuronal cells [7, 15, 18, 28–30]. During neuronal development, 5hmC not only displayed cell- and tissue-specific features but also displayed dynamic features in the genome [7, 18, 19]. 5hmC, 5fC, and 5caC were shown to be enriched in distinct genomic regions including promoters and enhancers [7, 31–33] and to display dynamic alteration during lineage specification [34, 35]. Thus, it is rational to speculate that the dynamic and specific distribution of active DNA demethylation could contribute to the proper gene expression during neuronal development.

Although 5fC and 5caC had been shown as stable epigenetic markers [36], their functions were still on the way to be explored. Genome-wide profiling studies revealed 5fC enriched in CpG islands (CGIs) of promoters, exons, and enhancers and involved in the regulation of gene expression during development [32, 37]. Recently, it was found that the level of 5caC increased in human breast cancer and during the differentiation of neural stem cells [34, 38]. Our present results indicated that 5fC and 5caC also exhibited dynamic features during the postnatal neuronal development. Together with these studies, 5fC and 5caC, like 5hmC, might also contribute to the regulation of cell- and region-specific gene expression.

In line with the temporospatial feature of DNA demethylation, the expression of Tets exhibited tissue-/cell-specific patterns and dynamic features during neuronal development [7, 19]. These findings suggested that despite having a similar structure, Tet1, 2, and 3 might have differential binding or catalytic activities towards the substrates, that is, 5hmC, 5fC, and 5caC [39]. The preference towards the differential substrates and distinct genomic loci could lead to the differential function. In the adult mouse brain, *Tet1* and *Tet2* deletion both regulated adult neurogenesis but displayed some differential effects [19, 20]. *Tet2* deletion preferentially induced 5hmC loss and hypermethylation at enhancers and gene bodies, but *Tet1* depletion predominantly reduced 5hmC levels at transcription start sites and promoter regions [19, 40, 41]. All these results suggested the distinct roles of *Tets* in different brain regions during development.

## Abbreviations

aNSC: Adult neural stem cell
Tet: Ten-eleven translocation
5mC: 5-Methylcytosine
5hmC: 5-Hydroxymethylcytosine
5fC: 5-Formylcytosine
5caC: 5-Carboxylcytosine
CTX: Cortex
HIPPO: Hippocampus
CER: Cerebellum.

## Supplementary Materials

Supplementary Materials Supplemental Figure 1: cultured NSCs express NSC markers and display the capabilities of self-renewal and multipotency. Cultured NSCs expressed the neural progenitor markers Sox2 and Nestin ((a)–(d)). Proliferative NSCs stained with BrdU ((e)–(g)). Cultured NSCs could differentiate into GFAP^+^ astrocytes and Tuj1^+^ neurons ((h)–(k)). Scale bar, 200 *μ*m.
