# Supplementary figures and images for: The Dynamic DNA Demethylation during Postnatal Neuronal Development and Neural Stem Cell Differentiation
Source: Stem Cells Int. 2018 Mar 11;2018:2186301. doi: 10.1155/2018/2186301 (PMC5866877; doi:10.1155/2018/2186301)

Supplemental Figure 1

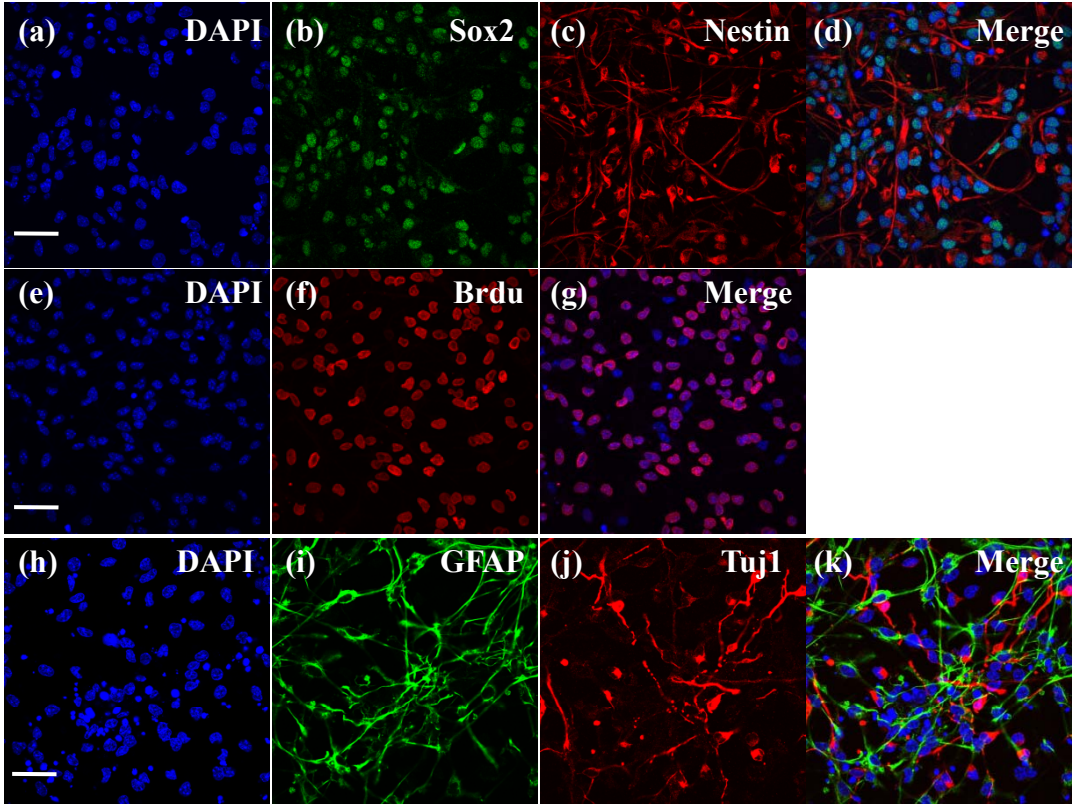

Supplement: Supplementary Materials — Supplemental Figure 1: cultured NSCs express NSC markers and display the capabilities of self-renewal and multipotency. Cultured NSCs expressed the neural progenitor markers Sox2 and Nestin ((a)–(d)). Proliferative NSCs stained with BrdU ((e)–(g)). Cultured NSCs could differentiate into GFAP+ astrocytes and Tuj1+ neurons ((h)–(k)). Scale bar, 200 μm. [file 2186301.f1.pdf]
